# Uncovering human *Dirofilaria repens* infections: new cases in Southern Italy

**DOI:** 10.1017/S0031182025000290

**Published:** 2025-04

**Authors:** Lavinia Ciuca, Simona Gabrielli, Patrizia Forgione, Evaristo Di Napoli, Orlando Paciello, Maria Vittoria Panariello, Marianna Ascierto, Luciana Petrullo, Maria Ortensia Montella, Maria Paola Maurelli, Laura Rinaldi

**Affiliations:** 1 Department of Veterinary Medicine and Animal Production, Center for Monitoring of Parasitosis (CREMOPAR), University of Naples Federico, Naples, Italy; 2 Department of Public Health and Infectious Diseases, University of Rome ‘Sapienza’, Rome, Italy; 3 Local Health Authority Naples 1 Center, Pellegrini Hospital, Naples, Italy; 4 Azienda of Colli of Naples, UOC Microbiology and Virology, Naples, Italy

**Keywords:** *Dirofilaria repens*-gravid females, epidemiology, human dirofilariosis, Southern Italy, zoonotic infection

## Abstract

*Dirofilaria repens* is the primary etiological agent of human dirofilariosis in the Old World, with Italy reporting the highest number of cases in Europe. This study describes two new cases of *D. repens* infection in humans, in southern Italy, where canine dirofilariosis is endemic. The first case involved a 33-year-old man from Caserta (Campania, Southern Italy) who presented with a subcutaneous mass on the upper eyelid. Surgical excision revealed an immature female *D. repens* worm measuring 14 cm, lacking microfilariae in both the uterus and peripheral blood. The second case was a 67-year-old man from Pozzuoli (Metropolitan City of Naples, Southern Italy) with an oval-shaped nodule in the left frontal scalp region. A live gravid female *D. repens* worm measuring 15–16 cm was extracted, also without microfilariae in the peripheral blood and no male worm detected. PCR sequencing confirmed a 100% match with *D. repens*. Both patients tested positive for *D. repens* antibodies by IgG ELISA. These cases underscore the continuous spread of human dirofilariosis in southern Italy and highlight diagnostic challenges due to variable clinical presentations. The discovery of a gravid female without microfilaremia suggests complexities in the parasite’s life cycle in humans, challenging the notion of humans as strict dead-end hosts. Given the rising prevalence in both humans and dogs, a comprehensive epidemiological study is recommended. Inclusion of dirofilariosis in the national surveillance system for notifiable diseases would improve case identification and tracking, aiding in better monitoring and control of this zoonotic infection.

## Graphical abstract

**Figure:**
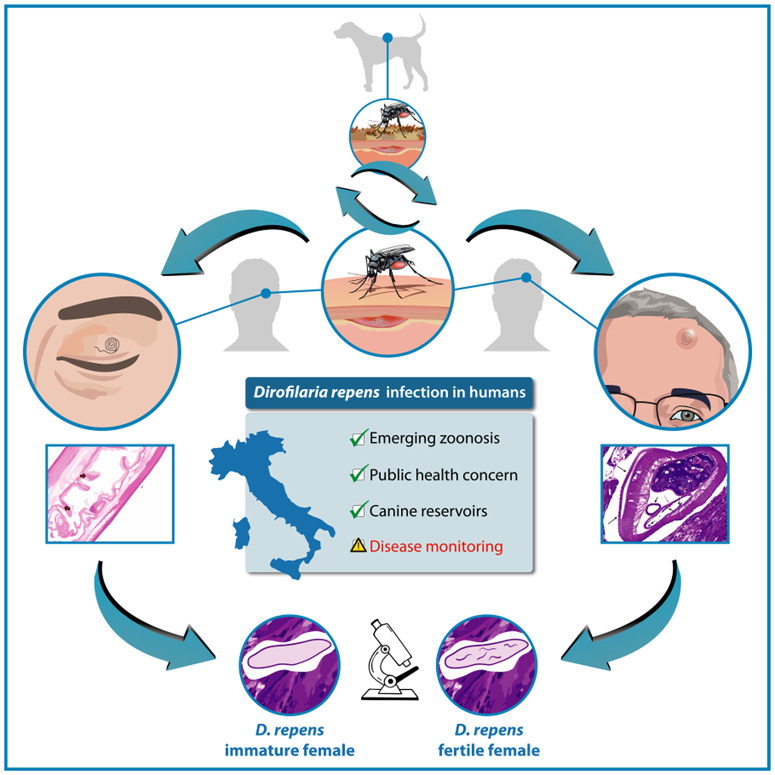

## Introduction

*Dirofilaria immitis* and *Dirofilaria repens* (Spirurida, Onchocercidae) are zoonotic vector-borne filarial parasites, transmitted by Culicidae mosquitoes, with dogs as the main reservoirs of infection (Capelli *et al*., 2018; Genchi and Kramer, 2020; Perles *et al*., 2024), in which the nematodes may cause heartworm disease or subcutaneous nodules, respectively (Simón *et al*., 2022). Data reported in recent studies shows an increase in the prevalence of both *D. immitis* and *D. repens* in Europe and in southeastern regions of Asia and Africa (McCall *et al*., 2008; Capelli *et al*., 2018; Genchi and Kramer, 2020). In Italy, the changing distribution patterns of both *D. immitis* and *D. repens* show an increase in their prevalence in dogs from areas considered non-endemic, such as regions in southern Italy (Genchi *et al*., 2009, 2019; Panarese *et al*., 2022; Ciucă *et al*., 2023; Napoli *et al*., 2023). Human infections are mainly due to *D. repens*, which remains a persistent public health problem (Szénási *et al*., 2008; Montoya-Alonso *et al*., 2010; Ciucă *et al*., 2018; Simón *et al*., 2022; Perles *et al*., 2024). Following transmission of the infecting larvae (L3) by mosquitoes, humans are usually accidental hosts for *D. immitis* and *D. repens*, although cases with fertile adults and/or microfilariaemia due to *D. repens* have been reported (Grandi *et al*., 2008; Poppert *et al*., 2009; Sergiev *et al*., 2009). Zoonotic infection results in the formation of nodular structures as a consequence of the circumscribed inflammatory response mounted around dead or dying larval stages. In most patients, *D. immitis* localizes in the lungs, causing pulmonary dirofilariosis, while *D. repens* localizes in the subcutaneous tissue – causing the formation of subcutaneous nodules – or under the conjunctiva (ocular dirofilariosis). Over the years, several cases of pulmonary nodules caused by *D. repens* have also been reported in the Old World (Pampiglione *et al*., 2009; Benzaquen *et al*., 2015; Gabrielli *et al*., 2021; Momčilović *et al*., 2022). Pulmonary dirofilariosis can be confused with pulmonary neoplasms (benign, carcinoma, metastases), tuberculosis or fungal infections (Awe *et al*., 1975; Ro *et al*., 1989; Oliva *et al*., 2019) and represents a diagnostic challenge (Simón *et al*., 2022). Between 1977 and 2016, more than 3500 human cases due to *D. repens* and 25 due to *D. immitis* were reported in Europe (Genchi and Kramer, 2017; Capelli *et al*., 2018). Since humans are not natural hosts, microfilariae are usually absent from peripheral blood. However, the parasite occasionally evades the host’s immune system to reach sexual maturity, resulting in microfilaremic infections that have so far been described in 23 human cases (Simón *et al*., 2022; Tasić-Otasevic *et al*., 2023). Italy is traditionally endemic for human dirofilariosis and is one of the countries with the highest number of human cases identified so far (Muro *et al*., 1999; Avellis *et al*., 2011; Capelli *et al*., 2018). In fact, the number of human cases of dirofilariosis caused by *D. repens* reported in the literature since Addario’s first observation in 1885 amounted to approximately 410 in 1995, 181 of which had occurred in Italy (Addario, 1885; Pampiglione and Rivasi, 2000). In 2001, there was a further description of 60 cases between 1990 and 1999 (Pampiglione *et al*., 2001). More recently, eight further cases of *D. repens* infection were described by Gabrielli *et al*. (2021). The present study aims to describe two new human cases of *D. repens* infection occurring in a region of southern Italy which has recently become endemic for canine dirofilariosis (Ciucă *et al*., 2023).

## Materials and methods

### Case histories and sampling of adult nematodes

#### First case (case 1)

**Figure 1.**
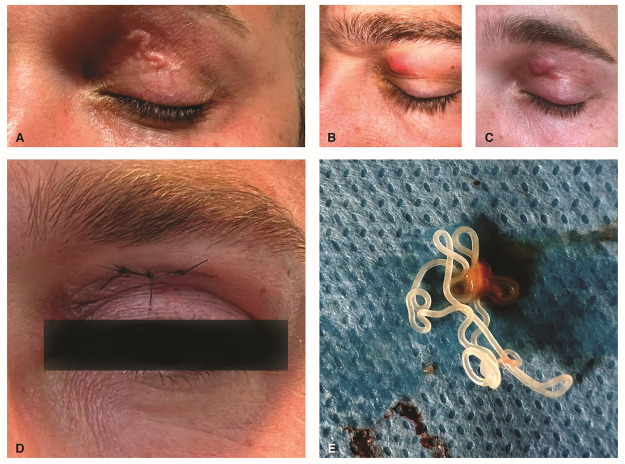
Case 1. (A) Patient with a serpiginous lump on the upper eyelid of the left eye at first presentation. (B, C) Patient with swelling of the upper eyelid of the left eye in the form of a lump after one week of treatment. (D) Patient after surgery. (E) Worm recovered after excision of the nodule from upper eyelid of the left eye.

In October 2023, a 33-year-old Italian-born man living in Caserta (Campania, southern Italy) was referred to the emergency hospital (Ospedale Dei Pellegrini) of Naples, with complaints of pruritus and swelling of the left upper eyelid (Figure 1A). In his description, he mentioned that he had been taking long walks along the river at Castel Volturno six months prior to his admission to the hospital. The man reported that he had been living with two dogs, aged 3 and 6 years old, for a period of 5 years. At the initial ophthalmological examination, the patient exhibited an oedema in a serpentiform shape on the upper eyelid of the left eye (Figure 1A). No evidence of swelling or similar rashes was observed in other parts of the body. The ophthalmological examination revealed that the visual acuity in both eyes was within the normal range. Given the possibility of a parasitic infection, the patient was treated with a dose of 12 mg of ivermectin. The following day, the patient was transferred to another hospital in Naples. Subsequently, he underwent the following additional diagnostic procedures: a chest X-ray, a CT scan, an ECG, blood tests, copromicroscopy examination and an IgG test for SARS-CoV-2. With the exception of the detection of *Blastocystis* spp. in the stool sample, all results were within the normal range. The patient was also examined by a dermatological specialist who prescribed a course of albendazole 400 mg twice a day, for 7 days. After treatment, the patient returned with a swelling of the upper eyelid in the form of a lump (Figure 1B,C). The snake-like appearance was no longer present. The patient was then referred for surgical excision of the nodule that had formed. A live and motile worm of approximately 14cm in length was found during the surgical excision of the nodule on the left upper eyelid (Figure 1D,E; supplementary file 1).

#### Second case (case 2)

**Figure 2.**
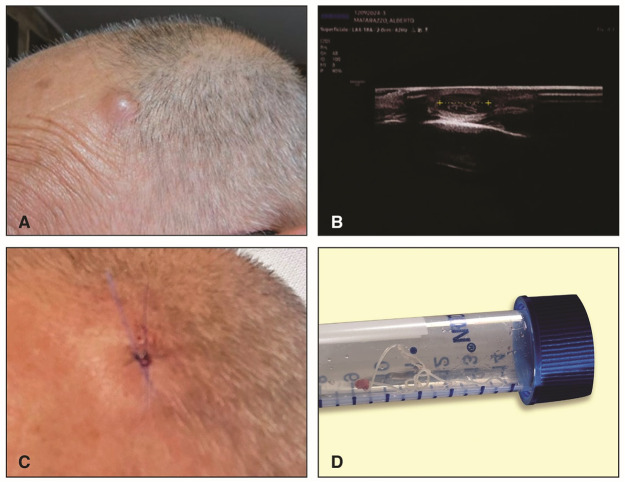
Case 2. (A) Patient with a subcutaneous mass located in the left frontal region of the scalp; (B) an ultrasound of the skin revealed an oval-shaped, hypoechoic nodule measuring 7.7 × 3.9 mm with well-defined, avascular margins in the scalp area, suggestive of the presence of a nematode; (C) patient after surgery; (D) a 10 cm-long nematode surgically removed from the nodule in the scalp area.

In September 2024, a 67-year-old Italian man from Pozzuoli, a coastal town in the Metropolitan City of Naples (southern Italy) was referred to a private dermatology clinic in Naples for an oval-shaped subcutaneous mass located in the left frontal region (Figure 2A). An ultrasound of the skin showed an oval-shaped nodule measuring 7.7 × 3.9 mm, hypoechoic with well-defined, avascular margins located in the scalp area (Figure 2B). Based on the ultrasound, a cyst or fibroma of the scalp was suspected, and surgical removal was recommended. During the surgical removal, a live worm measuring 10 cm in length was found (Figure 2C,D). The patient had no history of travel abroad in the past 3 years and owned an 18-month-old dog.

The 2 adult nematodes (the first along with the excised nodule from Case 1 and the second from Case 2), were sent to the Laboratory of Parasitology of the University of Naples Federico II, for histological and molecular analysis. Based on the final surgical results, there was a suspicion of infection with *Dirofilaria* spp. At this point, blood and serum samples were obtained from both patients to verify the presence of microfilariae in the blood and of specific antibodies against *Dirofilaria* spp. in the serum. Similarly blood and serum samples were collected from all dogs owned by both patients (2 from Case 1 and 1 from Case 2) and tested for the presence of microfilariae, *D. immitis* antigens and antibodies against *D. repens* somatic antigens.

The immunological tests were performed at the Department of Public Health and Infectious Diseases, University of Rome ‘Sapienza’, Rome, Italy.

### Laboratory investigation of the human cases: histology, molecular analysis, modified Knott’s test and immunological tests

The worms and nodule samples (Case 1 and Case 2) were excised, then preserved in neutral buffered formalin to maintain tissue integrity. After fixation, they were embedded in paraffin, allowing for thin slicing at 3–5 μm thickness for detailed microscopic analysis. The sections were then stained with haematoxylin and eosin, a standard method for distinguishing cellular and tissue structures due to haematoxylin staining nuclei blue and eosin staining the cytoplasm and extracellular matrix pink. Digital images were captured using a Pannoramic SCAN II (3DHISTECH) digital slide scanner, enabling detailed examination and production of representative images for further analysis.

For molecular identification, genomic DNA was extracted from 25 mg of tissue from each nematode, using the DNeasy® Blood and Tissue kit (Qiagen, Germany), following the manufacturer’s instructions. Molecular analyses were performed in accordance with the multiplex PCR protocol described by Rishniw *et al*. (2006) (5.8 + ITS2 region) which allows for the simultaneous detection of *D. immitis* and *D. repens.*

A modified Knott’s test was used for the detection of circulating microfilariae of *D. repens* (Genchi *et al*., 2021) as outlined below. One mL of EDTA blood (3× 1 mL for each tube) was mixed with 9 mL of distilled water and centrifuged for 3–5 min at 170 *g.* The supernatant was then removed from the tube and the contents were stained with 1–2 drops of 1% methylene blue. A drop was placed on a microscope slide covered with a cover slip and observed under an optical microscope at 100×.

The patient serum samples were analysed using an home-made ELISA test to detect the specific IgG antibody response. This involved the use of adult *D. immitis* and *D. repens* somatic antigens as described in Cabrera *et al*. (2018) and Mendoza-Roldán *et al*. (2021). The antigens were isolated from worms obtained by necropsy of naturally infected dogs, washed, and then they were macerated and sonicated (three cycles of 70 kHz, 30 s) in sterile saline solution (Prieto *et al*., 1997). The homogenate was centrifuged at 16 000*g* for 30 min. The supernatant was dialyzed against 0.01M PBS, pH 7.2. The protein concentration was measured (Bradford, 1976) and an ELISA microplate was coated with antigens at a final concentration of 0.8 µg/µL. The serum sample was tested in solid-phase ELISA at dilution 1:80 and 1:150 to detect anti-*D. repens* and anti-*D. immitis* IgG antibodies, respectively. Goat anti-human-IgG-antihuman IgG (H + L) conjugated to horseradish peroxidase (Sig-ma- Aldrich, MO, USA) was used as a secondary antibody at 1:40.000 dilution in both cases. The Optical density (OD) was determined at a wavelength of 492 nm (Easy-Reader, Bio Rad, CA, USA). The cut-off points (OD = 1.12 for DiSA and OD = 1.03 for DrSA) were established by calculating the mean value + 3 standard deviations (3SD) of 30 serum samples obtained from clinically healthy humans (negative controls).

### Laboratory investigations on the patients’ dogs

The dogs were screened for *Dirofilaria* spp. microfilariae using the Knott test (only 1 mL of EDTA blood) (Genchi *et al*., 2021). Additionally, the Petcheck Heartworm Canine (IDEXX) test was used according to the manufacturer’s instructions, to detect *D. immitis* antigen. All dogs were also screened for antibodies against *D. repens* (Mendoza-Roldán *et al*., 2021).

## Results

### Histology – case 1 and case 2

**Figure 3.**
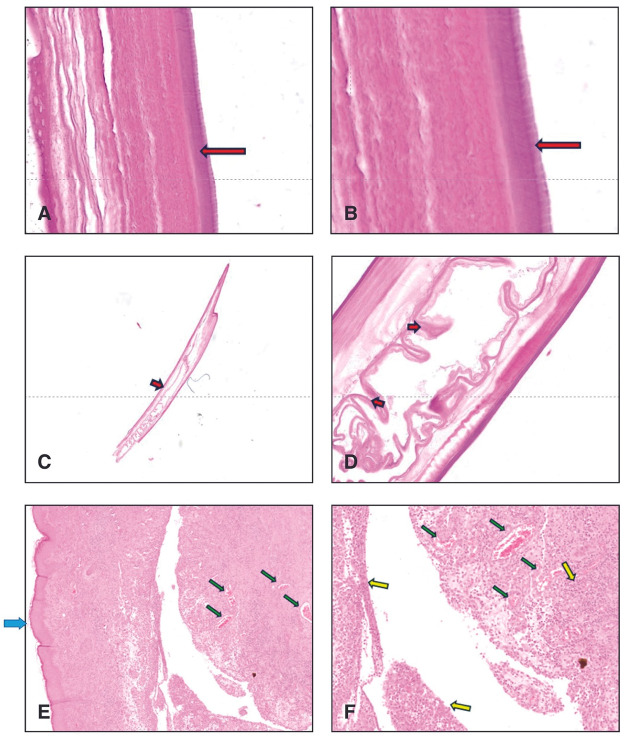
Hystological analysis case 1. (A,B) Longitudinal section of the parasite showing a thick, intensely eosinophilic cuticle covered with several layers of chitin raised in ridges (red arrows) (E.E. 40×); (C) longitudinal section of the parasite: caudal end. (E.E. 20×). (D) Longitudinal section of the parasite, coelomic cavity: a monolayer of cubic or cylindrical cells is observed (compatible with the intestine, red arrows). No microfilariae are observed. (E.E. 40×); (E,F) nodule. Cutis sections: the stratum corneum epithelium (blue arrow) is diffusely hyperplastic. The dermis is extensively expanded due to the presence of a chronic inflammatory infiltrate, mainly characterised by a high number of activated macrophages, epithelioid cells and eosinophilic granulocytes and a lower number of small mature lymphocytes and plasma cells (yellow arrows). There are multifocal areas of repair characterised by the presence of blood vessels (green arrows), fibroblasts associated with fibrillar matrix (fibroplasia) (E.E. 20×).

In the first case, the nematode displayed a whitish, cylindrical body measuring 14 cm in length. Histological characteristics were consistent with an immature female nematode (Wong and Brummer, 1978; Brindicci *et al*., 2019). Additionally, a thick, intensely eosinophilic cuticle was observed, covered with multiple chitinous layers forming ridges. This ridged appearance of the cuticle was characteristic of *D. repens* (Figure 3A,B). The nematode’s coelomic cavity contained a monolayer of cuboidal or cylindrical cells, consistent with intestinal tissue. No microfilariae were observed in the uterus (Figure 3C,D). Macroscopically, the nodule measured 1 × 1 cm, was spheroidal in shape, firm in consistency, and smooth on the surface. Histologically, the outer skin epithelium of the nodule was diffusely hyperplastic. The dermis was significantly expanded due to chronic inflammatory infiltrate, mainly comprising numerous activated macrophages, epithelioid cells, and eosinophilic granulocytes, with a smaller number of mature lymphocytes and plasma cells (Figure 3E,F).

**Figure 4.**
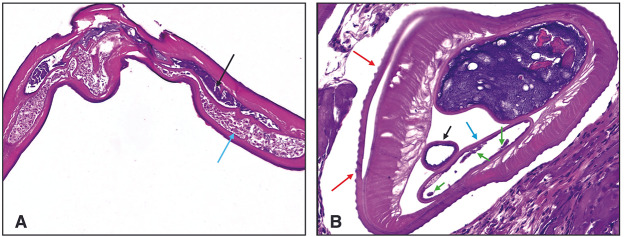
Hystological analysis case 2. (A,B) Longitudinal section of the parasite: intestine (black arrow) and uterus containing microfilariae (blue arrow) (E.E. 20×). Transverse section of the parasite: a thick, intensely eosinophilic cuticle is observed, covered by multiple layers of chitin raised into crests (red arrows), intestine (black arrow), and uterus (blue arrow) with microfilariae (green arrow) (E.E. 20×).

For the second case, the nematode displayed a whitish, cylindrical body and with ridged appearance of the cuticle measuring 15–16 cm in length. Histological characteristics were consistent with a fertile female nematode (Ciucă *et al*., 2020; Simón *et al*., 2022), showing in the transverse section the uterus with microfilariae (Figure 4A,B).

### Knott test and multiplex PCR (case 1 and case 2)

The modified Knott’s test revealed the absence of microfilariae in the blood of both patients.

The multiplex PCR method amplified a 484-base pair fragment specific to *D. repens* in both patients. The sequences of the PCR products obtained are available in the GenBank database under the following accession numbers: AY693808 (Case 1) and MW242631.1 (Case 2).

The first patient (Case 1) showed a positive response to *D. repens* somatic antigens (OD = 1.24) and a negative response to *D. immitis* (OD = 0.64) somatic antigens as determined by in-house IgG.

Similarly, the second patient (Case 2) was seropositive for *D. repens* (OD = 1.86) while no specific IgG were detected against *D. immitis* antigens (OD = 0.63).

### Test results for Dirofilaria spp in dogs

All dogs tested negative for *Dirofilaria* infection in both the modified Knott’s test and the antigen test. In addition, the ELISA IgG assay for antibodies against *D. repens* also yielded negative results for the three dogs tested.

## Discussion

The present study unveiled two new cases of human *Dirofilaria repens* infection in southern Italy and involved clinical, serological, haematological and molecular analysis in the two patients and their dogs, adopting an integrated medical and veterinary approach.

*Dirofilaria repens* is the main etiological agent of human dirofilariosis in the Old World (Genchi and Kramer, 2020). The rise in dirofilariosis cases across Europe has been linked to factors such as climate change, an increase in mosquito species capable of transmitting *Dirofilaria* spp., and the intensification of international dog trade and movement (Cancrini *et al*., 2007; Genchi *et al*., 2009; Capelli *et al*., 2013, 2018; Sałamatin *et al*., 2013; Simón *et al*., 2017; Perles *et al*., 2024). In Ukraine, for instance, dirofilariosis was included in the national surveillance system for notifiable diseases in 1975, and the country has thousands of cases reported in the archives of the Ministry of Health, even if not all cases are documented in international scientific journals (Sałamatin *et al*., 2013; ESDA, 2017; Simón *et al*., 2017, 2022).

Human dirofilariosis cases have been reported mainly from canine dirofilariosis endemic areas in the Mediterranean region (Capelli *et al*., 2018; Tahir *et al*., 2019), with Italy being the country with the highest number of reported cases to date (Pampiglione *et al*., 2001; Capelli *et al*., 2018; Gabrielli *et al*., 2021; Simon *et al*., 2022, 2024). Considering that Italy is an endemic country for canine and human dirofilariosis (Capelli *et al*., 2018; Genchi *et al*., 2019, 2023; Brianti *et al*., 2022; Napoli *et al*., 2023) not only is an increased number of cases of *D. immitis* and *D. repens* in both dogs and humans expected, but also a high frequency of aberrant migrations in humans, especially due to *D. repens*, as already shown in dogs (Pierantozzi *et al*., 2017; Napoli *et al*., 2024). As an example, *D. repens* infections of the male genitalia have been documented in humans (Pampiglione and Rivasi, 2000; Simón *et al*., 2022) including a case of testicular infection with *D. repens* recently reported in a child from northeastern Italy (Ugolini *et al*., 2022). Moreover, it should also be noted that two cases of *D. immitis* infection in humans, have already been reported in central Italy, one with localization in the right temporal bulbar subconjunctival space and the other with a 2 cm nodule in the right upper lung (Avellis *et al*., 2011; Palicelli *et al*., 2022).

The two human cases of *D. repens* infection reported in this study provide further insight into the epidemiological and clinical features of human dirofilariosis in an endemic region of southern Italy, where canine dirofilariosis is prevalent (Ferrara *et al*., 2022a; Ciucă *et al*., 2023; Napoli *et al*., 2023). Indeed, human dirofilariosis cases in southern Italy have been progressively increasing. The earliest reported cases date back to 1885, with 11 cases documented (Pampiglione *et al*., 2001). After a long hiatus, a new case emerged in an hospital in Naples in 2012: a 17-year-old male who presented with a pulmonary nodule that was initially suspected to be a lung tumour; histopathological examination confirmed the presence of *D. repens*, highlighting the diagnostic challenges posed by the atypical pulmonary location mimic malignancies (Gabrielli *et al*., 2012). In 2018, Ferrari *et al*. reported a rare case of pulmonary dirofilariosis caused by *D. repens* in a 62-year-old man from Palermo, who presented with pulmonary nodules that were initially suspected to be malignant based on imaging studies. Subsequently, in 2019, a case was reported in Bari, involving an 82-year-old woman with a subcutaneous *D. repens* lesion on her right thigh (Brindicci *et al*., 2019). Moreover, in 2022, it was reported a case of cutaneous dirofilariosis caused by *D. repens* in an Italian girl from Naples (southern Italy) who spends her summer holidays in the Tuscan countryside (northern Italy) (Ferrara *et al*., 2022b).

Our study describes two additional autochthonous cases of subcutaneous *D. repens* infections in southern Italy: a 33-year-old man from Caserta (Case 1), and a 67-year-old man from Pozzuoli, a coastal town in the Metropolitan City of Naples (Case 2). These cases evidence the continuous spreading of human dirofilariosis in southern Italy and the importance of accurate diagnostic approaches. Moreover this findings, alongside our cases of subcutaneous localization, reflects the diverse manifestations of *D. repens*, demonstrating its potential for internal organ involvement, albeit rarely, as also documented in literature (Ro *et al*., 1989; Ferrari *et al*., 2018; Oliva *et al*., 2019). Together, these cases highlight the need for awareness of atypical presentations of dirofilariosis in endemic regions, where superficial and deep tissue nodules may both occur.

Genetic analysis has shown considerable variability among isolates from both humans and dogs in Italy, indicating potential intraspecific diversity that may influence transmission dynamics and clinical manifestations (Genchi and Kramer, 2017). This genetic diversity could also explain the variety in human presentations, from superficial nodules to rare internal cases. Such variability underscores the importance of molecular diagnostics in distinguishing between different strains, as it may have implications for tracking infection sources and understanding regional differences in transmission and pathogenicity (Gabrielli *et al*., 2024). However, a major limitation of the present study is that the genetic variability of the strains in both cases was not characterized.

The distribution of patients by age and sex is not uniform. Most reported cases of *D. repens* infection occur in individuals aged 20–69, with the highest incidence observed in those aged 50–59 years and a greater prevalence in women than in men (Sałamatin *et al*., 2013; Gabrielli *et al*., 2021; Simón *et al*., 2022). In the current study, one patient (Case 2) falls within the age range with the highest incidence, while the other patient (Case 1) is within the broader 20–69 age range. Moreover, both patients were male, aligning with findings from other studies (Pampiglione *et al*., 1994; Pupić-Bakrač *et al*., 2021) and contrasting with the cases reviewed by Simón *et al*. (2022). Several studies have indicated that there is no statistically significant correlation between human subcutaneous dirofilariosis and either age or gender (Pampiglione *et al*., 2001; Fontes-Sousa *et al*., 2019; Pupić-Bakrač *et al*., 2021). Nevertheless, geographic variations in age distributions have been noted. For example, However, while cases in children are rare in Italy (Bertozzi *et al*., 2015; Pansini *et al*., 2022; Ugolini *et al*., 2022), in Sri Lanka it is common to find infected children under 5 years of age – possibly due to differences in clothing, customs, and vector preferences (Senanayake *et al*., 2013; Balendran *et al*., 2022) Additionally, a case in a 100-year-old individual is also unusual in Italy, perhaps reflecting the reduced effectiveness of insecticides in the Piedmont area (Pampiglione *et al*., 2001). Ultimately, the risk factors for this disease extend beyond age and gender and include the endemicity of the region (evidenced by infections in dogs and cats), areas with increased vector exposure, and lifestyle factors, such as herein presented, where both patients frequently took long walks in areas known to have canine cases. Other factors, such as individual immunological status, may also contribute, as suggested by other studies (Pupić-Bakrač *et al*., 2021; Simón *et al*., 2022).

Almost all human cases of *D. repens* reported in Italy have been asymptomatic or have presented with transient localized symptoms such as swelling, erythema, rash and pruritus (Rivasi *et al*., 2006; Ermakova *et al*., 2017; Gabrielli *et al*., 2021; Pupić-Bakrač *et al*., 2021). These symptoms are usually associated with subcutaneous or submucosal nodules in various parts of the body and can even involve the eye through the conjunctiva (Pampiglione *et al*., 1995, 2001; Pampiglione and Rivasi, 2000; Eccher *et al*., 2008; Palicelli *et al*., 2014; Fontanelli Sulekova *et al*., 2016). Only rarely the worm can migrate to deeper tissues such as muscles and lungs (Ermakova *et al*., 2017; Gabrielli *et al*., 2021; Pupić-Bakrač *et al*., 2021; Simón *et al*., 2022; Napolitano *et al*., 2023). The two cases in our study align with typical human presentations with subcutaneous nodules without blood microfilariae (Pampiglione and Rivasi, 2000; Pampiglione *et al*., 2001; Gabrielli *et al*., 2021). In Case 1, the patient presented with a subcutaneous mass in the upper eyelid, while Case 2 presented with an oval-shaped nodule in the left frontal region of the scalp. Furthermore, the nematode’s presence in the subcutaneous tissue of both cases ‘resembles *D. repens*’, as evidenced by similar cases from Italy (Brindicci *et al*., 2019; Gabrielli *et al*., 2021; Campana *et al*., 2022). Both cases in the present study showed infection sites similar to those commonly reported in a Ukrainian study, which analysed clinical data from 755 cases and found that 64.6% (488) of parasitic lesions were located in the head, including 297 cases around the eyes (Sałamatin *et al*., 2013). Additionally, the eyelid is the most commonly reported anatomical site for nodules caused by *D. repens*, as highlighted in the recent case review by Simón *et al*. (2022). This prevalence is likely because parasitic lesions in other areas are more difficult for patients to detect.

Humans are considered accidental hosts for *Dirofilaria* spp., as nematodes rarely reach sexual maturity or produce microfilariae in human blood (Simón *et al*., 2012; Ermakova *et al*., 2017). However, some studies have documented *D. repens* infections with microfilariae in blood or local tissues (Kłudkowska *et al*., 2018; Potters *et al*., 2018; Pupić-Bakrač *et al*., 2021; Tasić-Otasevic *et al*., 2023).

In our study, the first patient presented with an immature adult female *D. repens* worm measuring 14 cm in length, lacking microfilariae in both the uterus and the blood, but exhibiting the presence of antibodies in the serum sample. Similar characteristics – including sexually immature nematodes, absence of microfilariae, and a positive IgG immune response – were reported in some of the cases described in central Italy (Gabrielli *et al*., 2021).The second case was initially suspected to be a cyst or fibroma based on ultrasound findings but was later identified as a live 16–17 cm *D. repens* worm upon surgical excision, highlighting the challenge of distinguishing dirofilariosis from benign tumours. A similar case of subcutaneous *D. repens* infection in the scalp region of a patient, was also reported in Slovenia in 2017 (Kotnik *et al*., 2022).

Furthermore, in both cases of the present study, the worms were encapsulated within nodules. This finding is consistent with the global cases reviewed by Simón *et al*. (2022), who reported that 96.68% (408) of *D. repens* worms were encapsulated within nodules, while only 3.32% (14) were free.

An intriguing aspect of our study was the discovery of a gravid female *D. repens* within the nodule of the second case, although no microfilariae were present in the blood and no male worm was detected. While most nematodes isolated from humans are immature, several studies have documented mature *D. repens* females carrying microfilariae in their uterus without releasing them into the bloodstream (Supriaga *et al*., 2004; Poppert *et al*., 2009; Makaveev *et al*., 2022; Simón *et al*., 2022). Furthermore, Ermakova *et al*. (2017) reported sexually mature *D. repens* in 10.4% of nodules, suggesting that humans may not be a ‘dead-end’ host for this helminth. Even though the detection of *D. repens* microfilariae in the bloodstream suggests the probable presence of both male and female adult worms within the human host, the conclusive identification of both sexes in human cases has yet to be confirmed. Notably, the present case is the first molecularly confirmed instance in Italy since 1992 of a gravid female *D. repens* without the presence of microfilariae in either the peripheral blood or local tissues. In the case documented by Pampiglione *et al*. (1992), a 53-year-old woman from the Campania region (Salerno province) had a parasite located in the left submammary region. The detection of microfilariae in the uterus suggested that she was also harbouring a mature male worm, although none was identified. This finding implied the potential for microfilariae release into the bloodstream, yet microfilaremia was not observed. Conversely, Fontanelli Sulekova *et al*. (2016) reported a case in Central Italy where microfilariae were detected in a fine-needle aspirate of a subcutaneous nodule, despite the absence of microfilaremia in peripheral blood samples. The infection did not become fully patent, indicating that while microfilariae can be present in local tissues, they may not always enter systemic circulation or lead to a fully developed infection.

These cases highlight the variability in human *D. repens* infections and suggest that the parasite’s life cycle in humans may be more complex than previously thought. The presence of gravid females and tissue microfilariae without corresponding microfilaremia challenges the notion of humans as strict dead-end hosts and underscores the need for further research to understand the transmission dynamics and host–parasite interactions of *D. repens* in human infections.

A recent study reviewing *D. repens* cases in humans with local microfilaremia or blood microfilaremia in Europe from 1992 to 2021 identified 20 patients (Pupić-Bakrač *et al*., 2021). Only four of these patients had a history of chronic immune disorders, while the others were either immunocompetent or had no relevant medical data available. In our study, the two patients had no history of chronic immune disorders or immunodeficiency and exhibited haematological and biochemical parameters within normal ranges. In contrast, cases of *D. repens* with microfilariae in the bloodstream have been associated with the development of eosinophilia (Simón *et al*., 2012; Pupić-Bakrač *et al*., 2021).

The diagnosis of human dirofilariosis can be challenging and difficult to achieve. Most cases present with ‘silent signs’, absence of microfilariae, or are misinterpreted as malignant tumours (Capelli *et al*., 2018; Ferrari *et al*., 2018; Miterpáková *et al*., 2022; Simón *et al*., 2022). Diagnosis is primarily based on histopathological examination of excised nodules (Miterpáková *et al*., 2022; Simón *et al*., 2022). In most cases, similar to our cases, the nematodes are identified by the typical features of striated cuticle for *D. repens* and smooth for *D. immitis* (with ridges and striae only on the ventral surface of the male caudal extremity) (Wong and Brummer, 1978; Simón *et al*., 2012). However, due to the presence of immature nematodes in the histology of nodules, it is sometimes difficult to distinguish them from fully developed adults at the species level. Thus, molecular analysis is a better tool to diagnose *Dirofilaria* spp. in humans, but also to understand the hidden diversity of *Dirofilaria* spp. (Rossi *et al*., 2015; Palicelli *et al*., 2022).

Current serological tests for human dirofilariosis have limitations in sensitivity and specificity. Commercial ELISA kits, such as those from Bordier Affinity Products SA, detect antibodies against multiple filarial genera, including *Dirofilaria*, while home-made IgG ELISA tests target *D. immitis* and *D. repens*. These limitations are exemplified by the low OD values observed in our cases, possibly due to the immature worm in the first case. Despite this, serological tests can serve as preliminary epidemiological screening tools to assess human exposure risk, especially in regions endemic for canine dirofilariasis. Human immunological responses to *Dirofilaria* spp. correlate with high dirofilariosis prevalence in canine populations (Ciucă *et al*., 2018; Genchi and Kramer, 2020; Mendoza-Roldán *et al*., 2021; Brianti *et al*., 2022; Perles *et al*., 2024).

A persistent challenge in the diagnosis of human dirofilariosis, despite its worldwide endemic status, is the difficulty many physicians face in recognizing clinical cases. This lack of awareness often leads to delays in diagnosis as physicians do not initially consider dirofilariosis. Such diagnostic delays are noted in numerous reports not only in Italy but worldwide, where patients struggle with the infection for a long time and often require guidance and support from specialists in parasitology to obtain a diagnosis (Pampiglione and Rivasi, 2000; Ilyasov *et al*., 2013; Ahmed *et al*., 2022; Miterpáková *et al*., 2022; Simón *et al*., 2022). For example, in Case 1 from our study, the patient was treated with ivermectin and albendazole for one week after the initial evaluation and subsequently referred to another hospital for further assessment – without excising the nodule as a primary intervention or recognizing dirofilariosis as a possible cause. Similarly, in Case 2, the nodule was initially suspected to be cystic in nature. Consequently, the classical presentation of *D. repens* infection went unrecognized at first, even though Italy is an endemic area for dirofilariosis in both dogs and humans. In southern Italy in particular, the prevalence of both *D. repens* and *D. immitis* is increasing, with prevalence in shelter dogs ranging from 10% to 75% (Ferrara *et al*., 2022a; Ciucă *et al*., 2023; Napoli *et al*., 2023).

However, it should be noted that the dogs owned by both patients in this study tested negative for *D. repens* microfilariae. This finding indicates that the infections in humans were not directly linked to their own pets, despite close contact with potential reservoir hosts highlights the complexity of the transmission dynamics and the importance of considering the wider dog population in endemic areas as well as environmental exposure to the mosquito vectors such as *Aedes albopictus* and *Culex pipiens* (Capelli *et al*., 2018; Ferrara *et al*., 2022a).

In contrast to our study, a case of *D. repens* in subcutaneous tissue was described in Croatia in which the patients’ own dogs were microfilaremic (Pupić-Bakrač *et al*., 2021). This highlights the variability of zoonotic transmission dynamics and underlines the importance of comprehensive diagnostic approaches in both humans and their pets.

In conclusion, Italy is facing increasing prevalence rates of dirofilariosis in dogs (Capelli *et al*., 2013; Genchi and Kramer, 2020; Brianti *et al*., 2022; Ciucă *et al*., 2023; Napoli *et al*., 2023) and a steady increase incidence in humans, with the highest number of reported cases in Europe (Pampiglione *et al*., 2001; Rivasi *et al*., 2006; Gabrielli *et al*., 2021; Mendoza-Roldán *et al*., 2021; Pupić-Bakrač *et al*., 2021; Palicelli *et al*., 2022; Perles *et al*., 2024). Therefore, a comprehensive and harmonized epidemiological study of human exposure to *Dirofilaria* spp. in Italy would be advisable to demonstrate the need for its inclusion in national disease surveillance and to better monitor the increasing number of zoonotic cases.

## Supporting information

Ciuca et al. supplementary material Ciuca et al. supplementary material

## Supplementary material

The supplementary material for this article can be found at https://doi.org/10.1017/S0031182025000290.
